# Hybrid surgery in excision of a Shamblin II glomus tumor

**DOI:** 10.1590/1677-5449.012218

**Published:** 2019-02-12

**Authors:** Alexandre Campos Moraes Amato, Diego Daniel Gomes Ferreira, Fernanda Teixeira Fonseca da Silva, Marielly Ayako Uemura, Thais de Oliveira Stucchi, Ricardo Virgínio dos Santos

**Affiliations:** 1 Instituto de Medicina Avançada – Amato, Departamento de Cirurgia Vascular, São Paulo, SP, Brasil.; 2 Universidade de Santo Amaro – UNISA, Faculdade de Medicina, Departamento de Cirurgia Vascular, São Paulo, SP, Brasil.; 3 Universidade de Santo Amaro – UNISA, Faculdade de Medicina, São Paulo, SP, Brasil.

**Keywords:** glomus jugulare, carotid body tumor, paraganglioma, carotid artery diseases

## Abstract

Glomus tumors are rare benign neoplasms originating from paraganglionic cells of the neural crest developing in the adventitious layer of the vessel. They are nonencapsulated and highly vascularized. A 64-year-old female patient was identified with a hypervascularized glomus tumor measuring 5 cm, posterior to the left carotid bifurcation and contralateral carotid occlusion. We performed preoperative embolization via endovascular access followed by direct percutaneous puncture, guided by angiography, to fill the remaining area. After embolization, surgical excision of the tumor was performed with reduced bleeding and it was easier to find the cleavage planes to adjacent structures. At late follow-up, the patient is free from tumor recurrence. The tumor was classified as Shamblin II, measuring 4 to 6 cm with moderate arterial insertion. Through this double approach we observed a relative reduction in intraoperative bleeding and improved identification of the cleavage plane, facilitating excision and avoiding surgical clamping.

## INTRODUCTION

 Glomus tumors are rare benign neoplasms that originate from paraganglionic neural crest cells developing in the paravertebral region and can affect cervical blood vessels, cranial nerves, and the autonomic nervous system, with an incidence of 1:30,000. 1 ^,^ 2 This type of tumor accounts for 60-70% of paragangliomas of the head and neck, they are highly vascularized and, although well-delineated, they are not encapusulated and proximity with important structures, such as nerves, combined with dense vascularization make resection difficult. 2 ^-^ 4 Diagnostic suspicion is aroused by clinical assessment, with autonomic symptoms, and/or imaging exams such as angiotomography (gold standard), with presence of the lyre sign and intense vascularization at the lesion site, while Doppler ultrasonography shows a vascularized mass in the region of the carotid bifurcation. Additionally, angiotomography and magnetic resonance angiography can identify location, extent, correlations with adjacent structures, and the vascular nature of the tumor. 5 Diagnosis of this comorbidity is confirmed by immunohistochemical assay revealing the presence of several hormonal neuropeptides such as serotonin, gastrin, substance P, somatostatin, and neuron-specific enolase, the last of which is the most trustworthy enzyme for diagnosis. 

 A carotid glomus tumor is difficult to diagnosis, because of its slow growth and asymptomatic course. 1 ^,^ 2 Treatment is surgical and should be performed early and with embolization prior to surgery, in order to optimize the procedure by devascularization of the tumor. 6

## CASE DESCRIPTION

**Figure 1:**
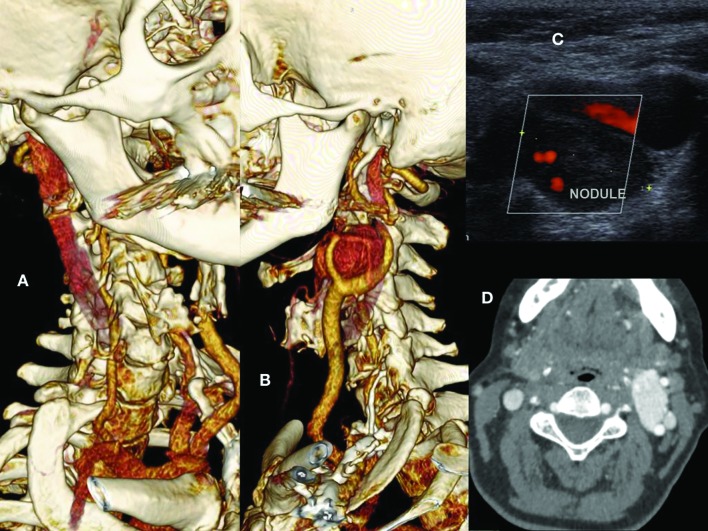
(A) 3D angiotomography reconstruction showing occlusion of the right carotid artery and vicarious vertebral artery; (B) 3D angiotomography reconstruction showing glomus tumor at the left carotid bifurcation; (C) Doppler ultrasonography showing a hypervascularized nodule between the left internal and external carotids; (D) Axial angiotomography slice showing hypervascularized glomus tumor on the left and carotid occlusion on the right.

 The patient was a 64-year-old female who sought care for a cervical nodule. Color Doppler ultrasonography revealed a large nodule posterior to the left carotid bifurcation and ligature of the right common carotid artery that had been performed during a previous surgical procedure. The patient was nevertheless asymptomatic neurologically. Arteriography ( Figure 1 A) identified a hypervascularized glomus tumor with a maximum diameter of 5 cm, located posterior to the left carotid bifurcation and primarily fed by the ascending pharyngeal artery, in addition to occlusion of the right carotid artery ( Figures 1 11D). Furthermore, a 4 mm saccular aneurysm was observed involving the left ophthalmic artery. The patient reported having had dermatological surgery previously in the right cervical area, which had involved complications causing her to be admitted to intensive care. However, she had no report or history providing details of that event. Having diagnosed the glomus tumor and contralateral carotid occlusion on the basis of imaging findings, the decision was taken to perform resection of the tumor after preoperative embolization. 

 Embolization was conducted by infusion of the Onyx^®^ copolymer embolic agent (Covidien, Irvine, CA, USA) 2 days before surgery, via superselective catheterization of the artery feeding the tumor, located at the carotid bifurcation. 

**Figure 2:**
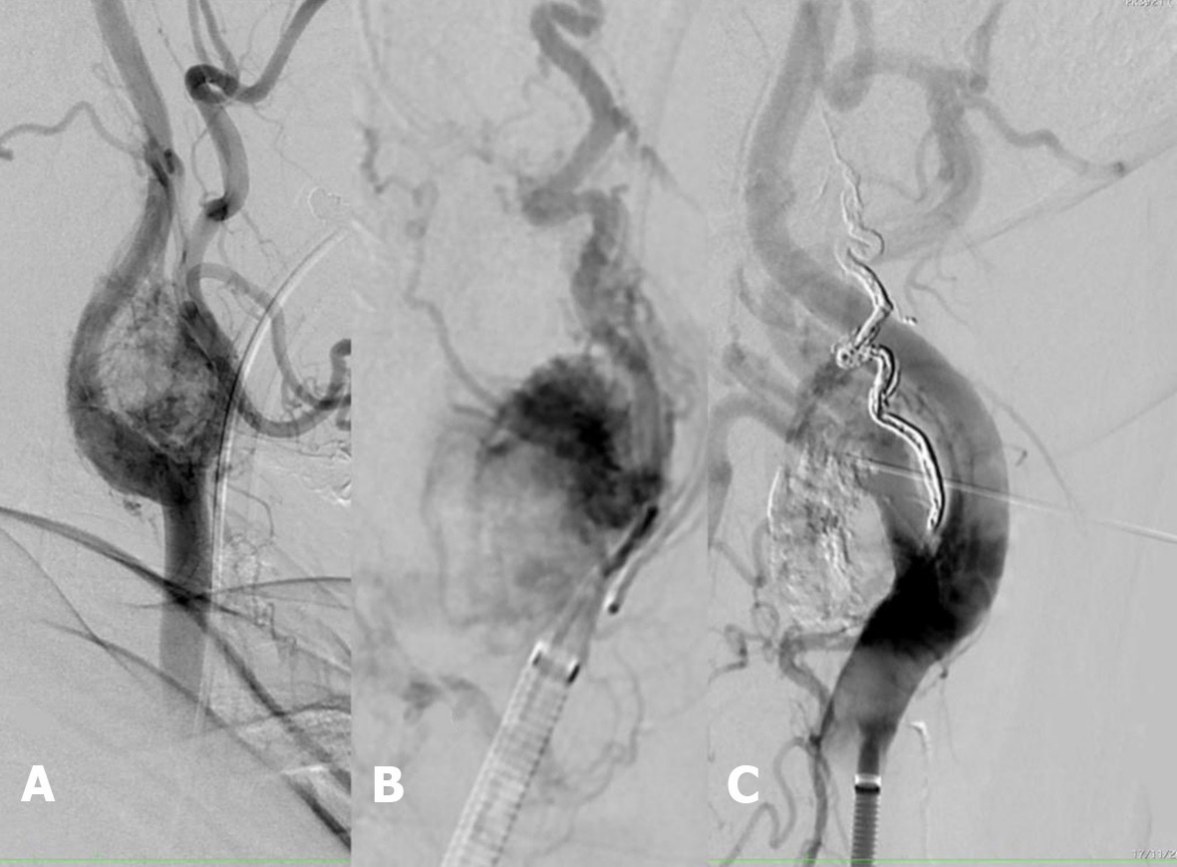
(A) Pre-embolization angiography showing the hypervascularized tumor and a left carotid lyre sign; (B) Superselective catheterization of the glomus tumor and “pressure cooker” embolization; (C) Direct external puncture of the glomus tumor and embolization.

**Figure 3:**
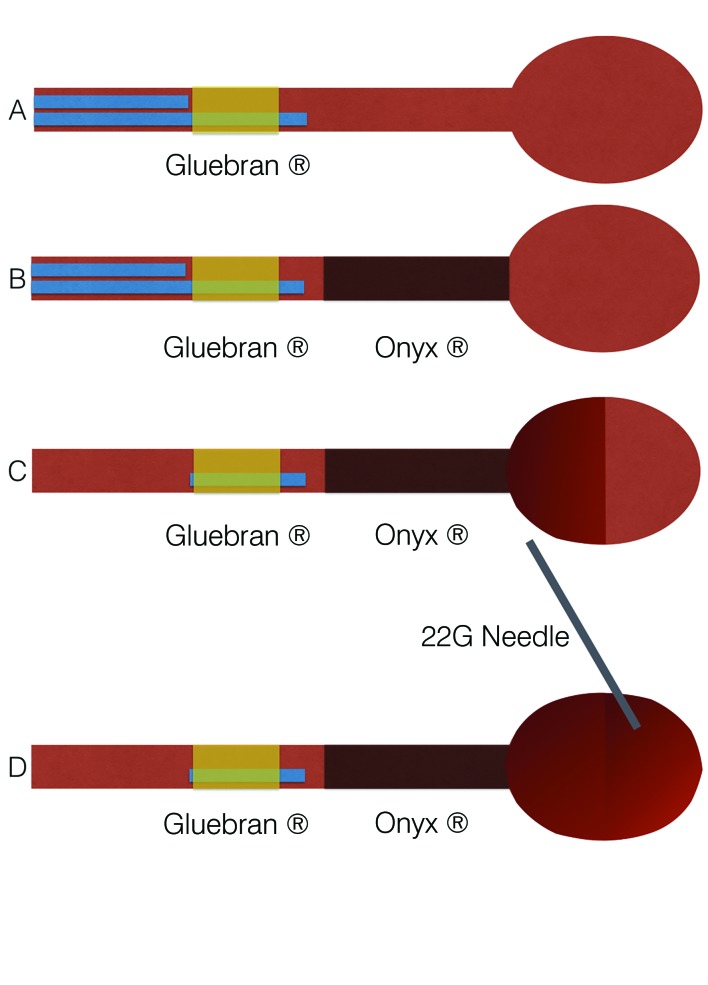
“Pressure cooker” embolization technique: (A) Advancing two microcatheters, one with a detachable tip, up to the artery feeding the tumor, and release of glue via the more distal catheter to secure the tip of the proximal catheter to the lesion, creating a “cork” effect; (B) Embolization of the vascularized tumor with Onyx^®^ via the catheter proximal of the lesion; (C) Withdrawal of both catheters. The tip of the more proximal catheter has been detached and glued where it remains inside the vessel; (D) Percutaneous puncture of the glomus tumor to fill the remaining area with polymer.

**Figure 4:**
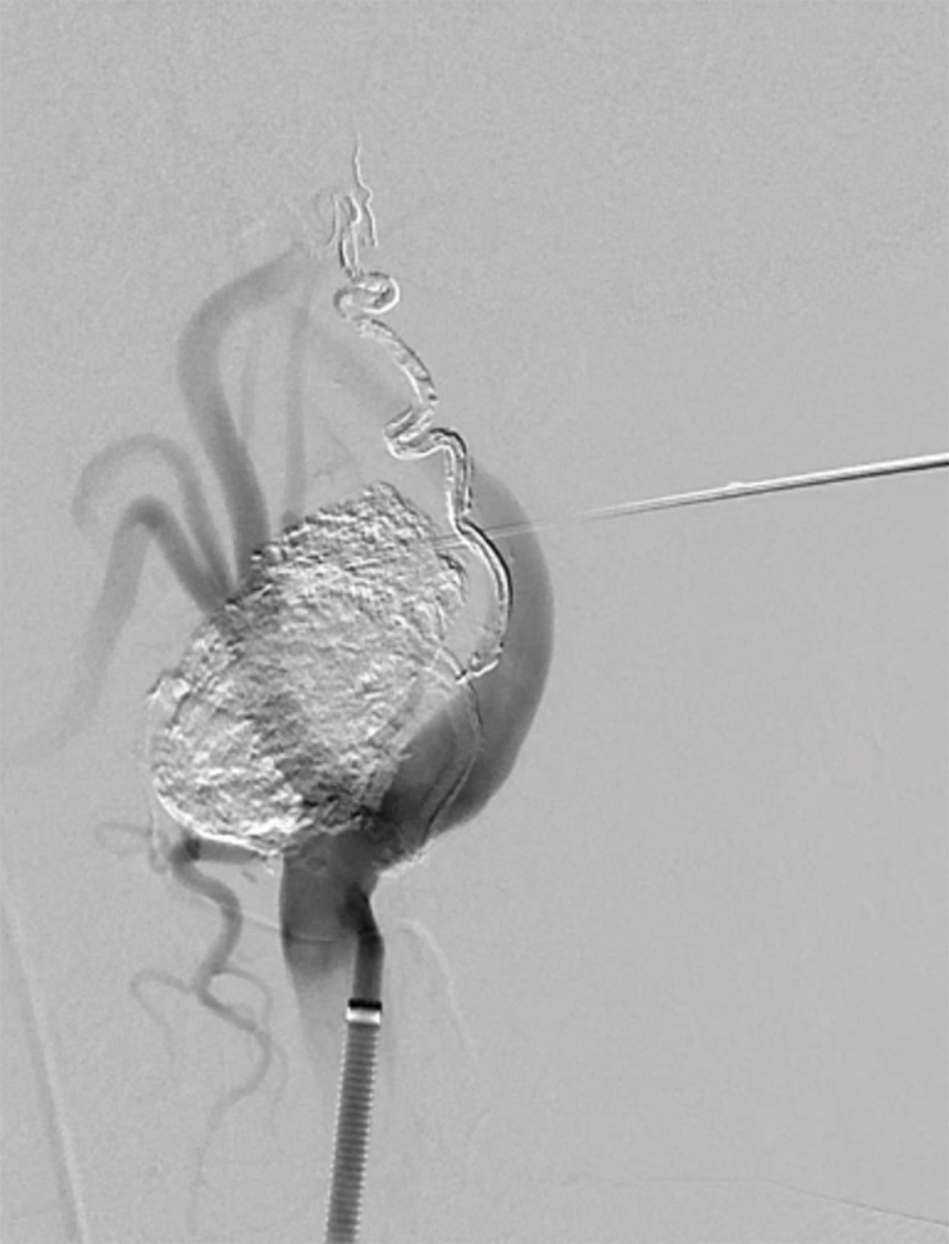
Arteriography showing the glomus tumor punctured percutaneously, soon after embolization with polymer. The arteriographic image is used to guide safe puncture of the tumor.

 The “pressure cooker” technique ( Figures 2 B and 3 ) was employed via a femoral artery puncture, with selective catheterization of the left common carotid artery ( Figure 2 A). Next, a 1.3-F Marathon^®^ microcatheter (Covidien, Irvine, CA, USA) was positioned in the ascending pharyngeal artery, occluding the proximal region. A 1.5-F Apollo ^®^ microcatheter (Covidien, Irvine, CA, USA) was then positioned distal of the Marathon^®^ catheter. The Apollo^®^ microcatheter has a mechanically detachable distal tip. It was used to inject Onyx^®^ until arterial reflux was identified on fluoroscopy ( Figure 2 B). Next, Gluebran^®^ was injected via the 1.3-F microcatheter to secure the tip of the Apollo® microcatheter and achieve arterial occlusion to prevent reflux into the carotid artery. Once the ascending pharyngeal artery had been obstructed, infusion of copolymer was resumed via the Marathon® catheter, to progress in the anterograde direction and fill the hypervascularized lesion ( Figures 3 33C). Since the hypervascularized tumor had not been completely filled ( Figure 3 C) by endovascular embolization, the unfilled part of the glomus tumor was directly punctured, percutaneously with a 22G needle, guided by the roadmap and fluoroscopy, and percutaneous polymer embolization was used to fill the remaining space ( Figures 2 C, 4 , and 3 D). 

**Figure 5:**
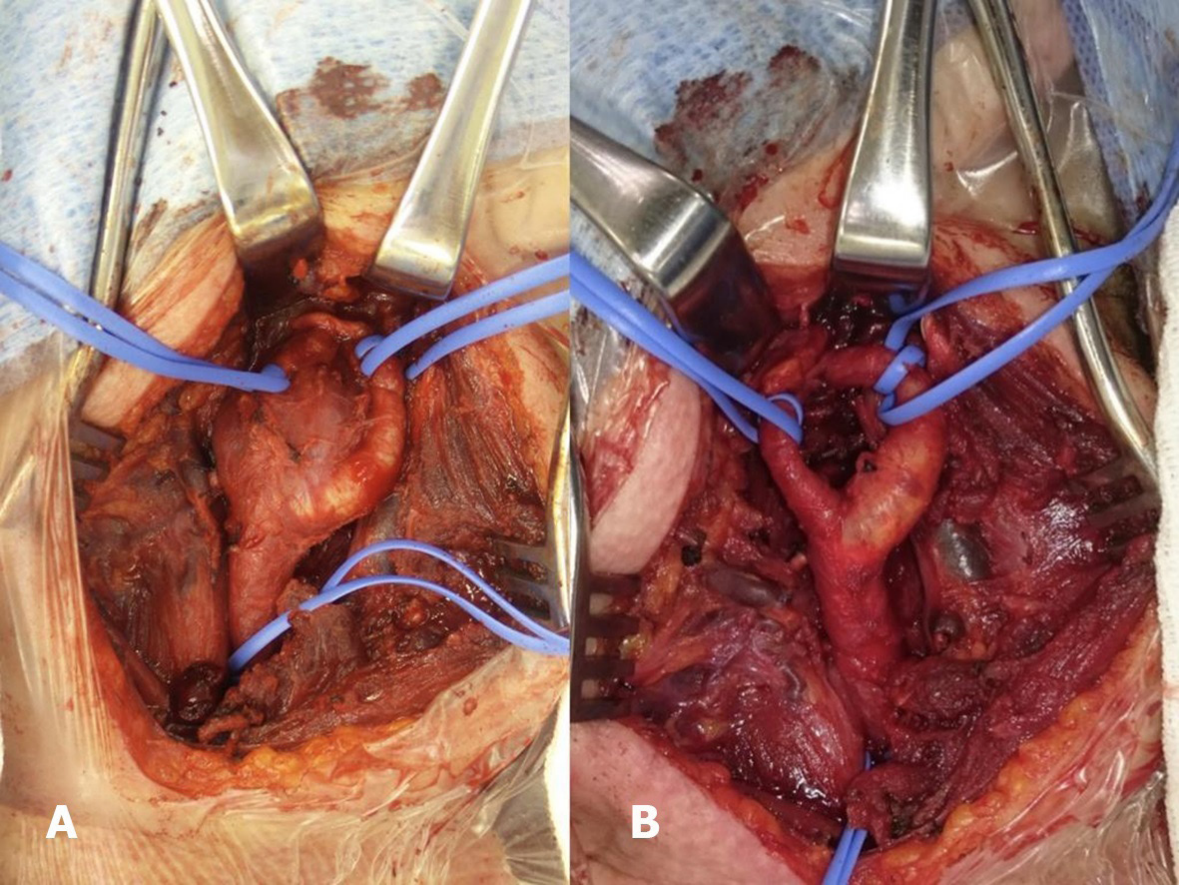
(A) Intraoperative image showing the glomus tumor adhering between the internal and external carotids; (B) Image after excision of the glomus tumor.

 Three days after embolization, surgical excision was performed through the longitudinal cervical incision typical of carotid endarterectomy, exposing an enlarged ganglion with a great deal of adherent tissues and signs of inflammation. After identification and isolation of arteries and nerves, a tumor with approximate dimensions of 3 cm x 2 cm was dissected without major bleeding or arterial damage, the dissection planes were identified, and rigorous hemostasis was achieved by ligation of the tumoral vessels and the ascending pharyngeal artery ( Figure 5 ). After excision, a soft silicone vacuum drain was attached and the dissection planes were drawn together. The results of pathology and immunohistochemical assays provided evidence of a paraganglioma with follicular lymphoid hyperplasia and reaction pattern. 

**Figure 6:**
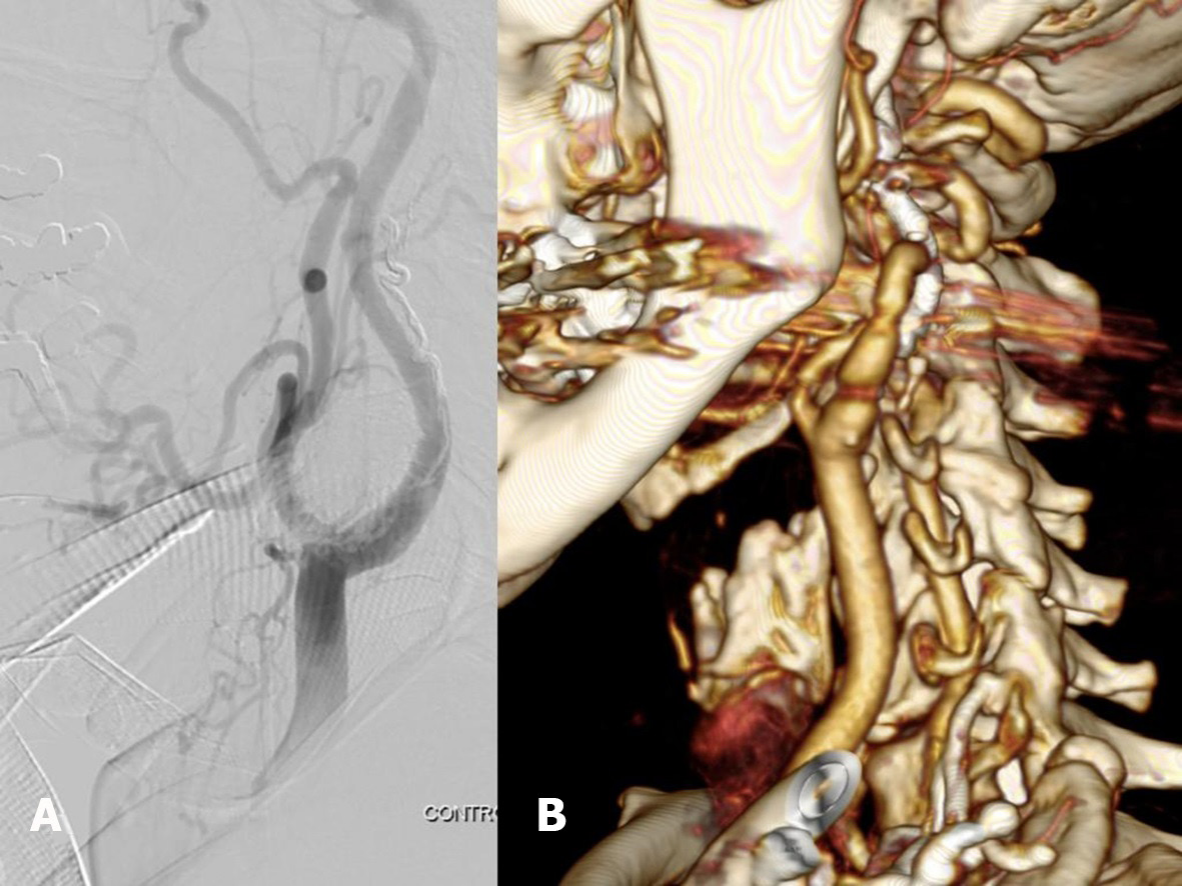
(A) Control angiography showing the final result of embolization; (B) Late 3D angiotomography reconstruction, 3 years after the surgical procedure.

 Initially, during the immediate postoperative period, systemic pressure was difficult to control, with hypertensive peaks, and intensive monitoring was needed for 5 days. After this period, the patient began to recover gradually, with a reduced need for antihypertensives, and was discharged 7 days after the surgical procedure. The late postoperative period went well, with no neurological events, good wound healing, no stenosis or expansive lesions, and no swellings. Imaging exams conducted as part of a reevaluation 3 years after the procedure did not show any sign of relapse whatsoever ( Figure 6 ). 

## DISCUSSION

 The diversity of glomus bodies in the human body means that tumors involving these structures can be found in many different sites. They have been reported in subungual regions, 7 ^,^ 8 and, more rarely, in other areas such as the liver, stomach, lungs, bones, joints, genital organs, central nervous system, and blood vessels. Carotid glomus tumors arise from paraganglion cells and develop in the adventitial layer of the vessel, at the bifurcation of the common carotid artery, in a posteromedial position. Although well-delineated, they are nonencapsulated tumors that are highly vascularized by branches of the external carotid artery and its vasa vasorum. 3 ^,^ 4 ^,^ 9 ^-^ 11

 Generally, the initial manifestation reported by patients is presence of a painless mass palpable in the lateral neck. Imaging exams such as computed tomography and color Doppler ultrasonography of the cervical region will show the tumor and its limits, providing information for staging and helping with choice of treatment. After a series of case studies, in 1971, Shamblin et al *.* published a classification of glomus tumors, categorized by size and degree of invasion of the vessel wall. 12 ^,^ 13 Tumors classified as belonging to group I are relatively small (< 4 cm) and minimally linked to the carotid vessels and can therefore generally be resected without difficulty. Group II encompasses larger neoplasms (4-6 cm) with moderate arterial insertion. Tumors classified as group III are large (> 6 cm) and imprison the carotids, requiring resection en bloc with the carotid bifurcation and revascularization with saphenous vein or prostheses. 13 ^,^ 14 The tumor in the case described here was classified as belonging to group II, because of the size of the neoplasm and its intermediate degree of adherence, but with an additional degree of difficulty caused by the contralateral carotid occlusion. 

 In 1980, Schick et al. reported the first successful embolization of a glomus tumor followed by surgical removal. They embolized the occipital and posterior auricular arteries and the thyrocervical trunk; post-embolization arteriography showed a 30% reduction in tumor size. 15 ^,^ 16 As an alternative to arterial embolization, studies have demonstrated that direct puncture of the hypervascularized tumor can also be considered safe. 17 Although embolization is not a definitive treatment, the literature indicates that removal of the tumor is more precise after embolization, because of devascularization of the neoplasm, reducing intraoperative bleeding, which improves the ability to see adjacent structures, avoiding damage to them and revealing the cleavage plane. While subjective, reduced intraoperative bleeding and easier dissection were noted by the surgeons. Sahin et al. performed a study with 12 patients operated for tumors of the carotid body (five Shamblin I and seven Shamblin II) who underwent preoperative direct percutaneous embolization, enabling better excision. 12

 Carotid glomus tumors are rare, hypervascularized, benign neoplasms that in certain cases cause compressive symptoms. Management is a technical challenge because of the size, location, and vascularization. Preoperative embolization of Shamblin II tumors can therefore be considered beneficial, since it reduces tumor vascularization. In the case described here, the strategy adopted avoided carotid clamping, preventing temporary cerebral ischemia and enabling the surgical technique to be performed safely, following the cleavage planes. This double approach reduced bleeding and facilitated excision of the tumor, thereby reducing surgical risk and achieving a satisfactory postoperative outcome. 

 Nevertheless, the decision to employ embolization should be made on a case-by-case basis, according to patient needs, taking into consideration risks and benefits, in addition to the experience of the professionals who will perform the procedure. 
