# Supplementary material for: Hybrid surgery in excision of a Shamblin II glomus tumor
Source: J Vasc Bras. 2019 Feb 12;18:e20180122. doi: 10.1590/1677-5449.012218 (PMC6579522; doi:10.1590/1677-5449.012218)
Supplement: Translation

# Translation: Cirurgia híbrida na exérese de tumor glômico Shamblin II

**Authors:** Alexandre Campos Moraes Amato, Diego Daniel Gomes Ferreira, Fernanda Teixeira Fonseca da Silva, Marielly Ayako Uemura, Thais de Oliveira Stucchi, Ricardo Virgínio dos Santos

**Affiliations:** 1 Instituto de Medicina Avançada – Amato, Departamento de Cirurgia Vascular, São Paulo, SP, Brasil.; 2 Universidade de Santo Amaro – UNISA, Faculdade de Medicina, Departamento de Cirurgia Vascular, São Paulo, SP, Brasil.; 3 Universidade de Santo Amaro – UNISA, Faculdade de Medicina, São Paulo, SP, Brasil.

**Keywords:** glomo jugular, tumor do corpo carotídeo, paraganglioma, doenças das artérias carótidas

## Abstract

O tumor glômico é uma neoplasia benigna rara originada de células paraganglionares da crista neural que se desenvolve na camada adventícia do vaso. São tumores não encapsulados e altamente vascularizados. Paciente feminina, 64 anos, foi diagnosticada com tumor glômico hipervascularizado com 5 cm posteriormente à bifurcação carotídea esquerda e oclusão de carótida contralateral. Optou-se por realizar embolização através de acesso endovascular seguida de punção percutânea direta, guiada por angiografia, para preenchimento da área remanescente. Após embolização, realizou-se a exérese cirúrgica do tumor com menor sangramento e maior facilidade para encontrar o plano de clivagem das estruturas adjacentes. Em acompanhamento tardio, a paciente apresenta-se sem recidiva tumoral. O tumor foi classificado como pertencente ao grupo Shamblin II, o qual inclui tumores apresentando de 4 a 6 cm com inserção arterial moderada. Através dessa dupla abordagem, foi possível notar uma redução relativa do sangramento intraoperatório e facilitação de identificação do plano de clivagem, colaborando para sua exérese e evitando o pinçamento cirúrgico.

## INTRODUÇÃO

 O tumor glômico é uma neoplasia benigna rara originada de células paraganglionares da crista neural e desenvolve-se na região paravertebral, podendo afetar vasos sanguíneos cervicais, nervos cranianos e o sistema nervoso autônomo, com incidência de 1:30.000 1 ^,^ 2 . Esse tumor representa 60-70% dos paragangliomas de cabeça e pescoço, é altamente vascularizado e, embora seja bem delimitado, não é encapsulado; além disso, a proximidade com estruturas importantes como nervos e a vascularização intensa dificulta sua ressecção 2 ^-^ 4 . A suspeita diagnóstica ocorre através da avaliação clínica, com alterações autonômicas, e/ou de exames de imagem como angiotomografia (padrão ouro), com a presença do sinal da lira e intensa vascularização no local da lesão, e eco-Doppler evidenciando uma massa vascularizada na região da bifurcação carotídea. Além disso, a angiotomografia e a angiorressonância identificam a localização, a extensão, a correlação com estruturas adjacente e a natureza vascular do tumor 5 . A confirmação dessa comorbidade é realizada através de exame imuno-histoquímico revelando a presença de múltiplos neuropeptídeos hormonais como serotonina, gastrina, substância P, somatostatina, e a enolase neurônio-específica, sendo esta última a enzima mais fidedigna para o diagnóstico. 

 O tumor glômico carotídeo é de difícil diagnóstico, devido ao seu lento crescimento e à sua progressão assintomática 1 ^,^ 2 . O tratamento é cirúrgico e deve ser realizado de forma precoce e associado a embolização pré-cirúrgica, a fim de otimizar o procedimento através da desvascularização do tumor 6 . 

## DESCRIÇÃO DO CASO

 Paciente do sexo feminino, 64 anos, queixando-se de nódulo cervical, apresentou ao eco-Doppler colorido um nódulo grande posterior à bifurcação carotídea esquerda e uma ligadura da artéria carótida comum direita realizada em procedimento cirúrgico prévio; apesar disso, a paciente era assintomática neurologicamente. Foi realizada arteriografia ( Figura 1 A), que identificou um tumor glômico hipervascularizado medindo 5 cm em seu maior diâmetro, de localização posterior à bifurcação carotídea esquerda e nutrido preferencialmente pela artéria faríngea ascendente, concomitante com oclusão de artéria carótida direita ( Figuras 1 11D). Além disso, foi observado um aneurisma sacular de 4 mm na artéria oftálmica esquerda. A paciente relatou cirurgia cervical dermatológica prévia à direita, em que teve complicações e ficou internada em regime de terapia intensiva; porém, não possuía relatório ou histórico detalhado do evento. Com o diagnóstico imageológico de tumor glômico e oclusão carotídea contralateral, optou-se pela ressecção do tumor precedida por embolização pré-operatória. 

**Figura 1:** (A) Reconstrução 3D de angiotomografia evidenciando oclusão de carótida direita e artéria vertebral vicariante; (B) Reconstrução 3D de angiotomografia evidenciando tumor glômico à esquerda em bifurcação carotídea; (C) Eco-Doppler mostrando nódulo hipervascularizado entre carótida interna e externa esquerda; (D) Corte axial de angiotomografia evidenciando tumor glômico hipervascularizado à esquerda e oclusão de carótida à direita.

 Foi realizada embolização com infusão do agente embólico/copolímero Onyx^®^ (Covidien, Irvine, CA, USA) 2 dias antes da cirurgia, por cateterização superseletiva da artéria nutridora do tumor, localizada na bifurcação carotídea. 

 A técnica da “panela de pressão” ( Figuras 2 B e 3 ) foi realizada através da punção da artéria femoral, sendo cateterizada seletivamente a artéria carótida comum esquerda ( Figura 2 A). Em seguida, posicionou-se um microcateter 1.3-F Marathon^®^ (Covidien, Irvine, CA, USA) na artéria faríngea ascendente, ocluindo-a na região proximal. Distalmente a esse microcateter, foi posicionado outro microcateter 1.5-F Apollo ^®^ (Covidien, Irvine, CA, USA), que tem como característica o destacamento mecânico de sua ponta distal. A partir deste último, foi realizada a injeção de Onyx^®^ até a ocorrência de refluxo arterial identificado na fluoroscopia ( Figura 2 B). Em seguida, foi injetada cola Gluebran^®^ pelo microcateter 1.3-F para fixação da ponta do microcateter Apollo® e oclusão arterial para evitar o refluxo para artéria carótida. Assim, com a obstrução da artéria faríngea ascendente, foi retomada a infusão do copolímero pelo cateter Marathon® para progressão anterógrada e preenchimento da lesão hipervascular ( Figuras 3 33C). Como não houve preenchimento completo do tumor hipervascularizado ( Figura 3 C) após a embolização endovascular, foi realizada punção percutânea direta do tumor glômico em área não preenchida, com agulha 22G, guiada por *roadmap* e fluoroscopia, sendo realizada nova embolização de polímero, agora percutânea direta, preenchendo os espaços remanescentes ( Figuras 2 C, 4 e 3 D). 

**Figura 2:** (A) Angiografia pré-embolização evidenciando tumor hipervascularizado e sinal da lira em carótida esquerda; (B) Cateterização superseletiva de tumor glômico e embolização pela técnica da “panela de pressão”; (C) Punção direta externa de tumor glômico com embolização.

**Figura 3:** Técnica da “panela de pressão” para embolização: (A) Passagem de dois microcateteres, sendo um de ponta destacável até artéria nutridora do tumor, e liberação de cola pelo cateter mais distal para fixação da ponta do cateter proximal à lesão e criação de efeito “rolha”; (B) Embolização de tumor vascularizado com Onyx ^®^ através de cateter proximal à lesão; (C) Retirada de ambos cateteres, sendo que a ponta do cateter mais proximal à lesão, fixada pela cola, é destacada e permanece no vaso; (D) Punção percutânea de tumor glômico com preenchimento de área restante por polímero.

**Figura 4:** Arteriografia evidenciando tumor glômico puncionado percutaneamente, logo após embolização com polímero. A imagem arteriográfica é utilizada como guia para punção segura do tumor.

 Três dias após a embolização, procedeu-se com exérese cirúrgica, que ocorreu através de uma incisão cervical longitudinal esquerda típica de endarterectomia carotídea, a partir da qual foi possível identificar gânglio aumentado de tamanho e tecidos muito aderidos, com sinais inflamatórios. Com a identificação e o isolamento de artérias e nervos, o tumor com aproximadamente 3 cm x 2 cm foi dissecado sem grandes sangramentos ou lesões arteriais, foram encontrados os planos de dissecção, e foi realizada uma hemostasia rigorosa com ligaduras de vasos tumorais e de artéria faríngea ascendente ( Figura 5 ). Após a exérese, fixou-se um dreno a vácuo de silicone maleável e realizou-se a aproximação dos planos de dissecção. O exame anatomopatológico, em conjunto com a avaliação imuno-histoquímica, evidenciou paraganglioma e hiperplasia linfoide folicular de padrão reacional. 

**Figura 5:** (A) Imagem intraoperatória mostrando tumor glômico aderido entre carótida interna e externa; (B) Imagem após exérese de tumor glômico.

 Inicialmente, no pós-operatório imediato houve um descontrole pressórico sistêmico com picos hipertensivos, sendo necessária monitorização intensiva por 5 dias. Após esse período, a paciente apresentou melhora gradual, com redução da necessidade de anti-hipertensivos, recebendo alta 7 dias após o procedimento cirúrgico. Seu pós-operatório tardio mostrou-se bom, sem eventos neurológicos, com boa cicatrização, sem estenose ou lesões expansivas, assim como ausência de abaulamentos. Em reavaliação após 3 anos do procedimento, exame de imagem não mostrou qualquer sinal de recidiva ( Figura 6 ). 

**Figura 6:** (A) Angiografia de controle evidenciando resultado final da embolização; (B) Reconstrução 3D de angiotomografia tardia após 3 anos do procedimento cirúrgico.

## DISCUSSÃO

 Devido à diversidade de corpos glômicos em nosso organismo, os tumores dessas estruturas podem ser encontrados em variados locais, sendo relatados na região subungueal 7 ^,^ 8 e mais raramente em outras regiões como fígado, estômago, pulmões, ossos, articulações, órgãos genitais, sistema nervoso central e vasos sanguíneos. O tumor glômico carotídeo surge de células paraganglionares e desenvolve-se na camada adventícia do vaso, na bifurcação da artéria carótida comum, em localização posteromedial. Embora sejam bem delimitados, são tumores não encapsulados e altamente vascularizados por ramos da artéria carótida externa e seus *vasa vasorum* 3 ^,^ 4 ^,^ 9 ^-^ 11 . 

 Geralmente, os pacientes relatam a presença de massa indolor palpável na região lateral do pescoço como manifestação inicial. Exames de imagem como tomografia computadorizada e eco-Doppler colorido da região cervical mostram o tumor e seus limites, fornecendo informações sobre o estadiamento e auxiliando na escolha do tratamento. Após uma série de estudos de casos, em 1971, Shamblin et al*.* publicaram uma classificação para os tumores glômicos de acordo com o seu tamanho e a sua invasão na parede do vaso 12 ^,^ 13 . Tumores classificados como pertencentes ao grupo I são relativamente pequenos (< 4 cm) e minimamente ligados aos vasos carotídeos; portanto, comumente podem ser ressecados sem dificuldades. O grupo II engloba neoplasias maiores (4-6 cm) que apresentam inserção arterial moderada. Os tumores classificados como grupo III são grandes (> 6 cm) e encarceram as carótidas, sendo necessária a ressecção em bloco com bifurcação carotídea e a realização de revascularização com veia safena ou prótese 13 ^,^ 14 . No caso apresentado, o tumor foi classificado como pertencente ao grupo II, devido ao tamanho da neoplasia e ao seu grau intermediário de aderência, porém com a dificuldade adicional da oclusão de carótida contralateral. 

 Em 1980, Schick et al. relataram a primeira embolização bem sucedida de um tumor glômico seguida por remoção cirúrgica. Foi realizada embolização das artérias auriculares occipital e posterior e do tronco tireocervical; a arteriografia pós-embolização demonstrou 30% de diminuição no tamanho do tumor 15 ^,^ 16 . Como alternativa à embolização arterial, estudos demonstram que a punção direta de tumoração hipervascularizada também é considerada segura 17 . Embora a embolização não seja um tratamento definitivo, a literatura aponta que a retirada do tumor torna-se mais precisa após a embolização, devido à desvascularização da neoplasia, diminuindo o sangramento intraoperatório, fato que possibilita uma melhor visualização das estruturas adjacentes, evitando lesá-las e evidenciando o plano de clivagem. A diminuição do sangramento intraoperatório e a maior facilidade de dissecção, embora subjetiva, foi notada pelos cirurgiões. Sahin et al. realizaram um estudo com 12 pacientes operados para tumor do corpo carotídeo (cinco Shamblin I e sete Shamblin II) que foram submetidos a embolização pré-operatória percutânea direta permitindo uma melhor exérese 12 . 

 O tumor glômico carotídeo representa uma neoplasia benigna rara hipervascularizada, que em alguns casos apresenta sintomas compressivos, sendo sua abordagem um desafio técnico, devido ao seu tamanho, localização e vascularização. Portanto, a embolização prévia do tumor Shamblin II pode ser considerada benéfica, devido ao decréscimo da vascularização do tumor. Neste relato de caso, a estratégia adotada evitou o pinçamento carotídeo, prevenindo isquemia cerebral temporária e permitindo a execução segura da técnica cirúrgica em tumor aderido aos planos de clivagem. A dupla abordagem reduziu sangramentos e facilitou a exérese tumoral, diminuindo assim os riscos cirúrgicos e tornando o pós-operatório satisfatório. 

 Porém, a decisão de embolização deve ser individualizada de acordo com as necessidades do paciente, levando em consideração riscos e benefícios, além da experiência dos profissionais que irão participar do procedimento.
